# Hospital Meetings, &c.

**Published:** 1905-02-11

**Authors:** 


### HOSPITAL MEETINGS, &c.

### CITY OF LONDON TRUSS SOCIETY.

The annual general meeting of the City of London Truss Society took place on Wednesday, 1st inst., at 35 Finsbury Square, E.C., Mr. Walter K. Taunton presiding.

The Chairman, in moving the adoption of the report and accounts for 1904, said that considering the difficulties in obtaining subscriptions to charities generally at the present time, the society might congratulate itself on the fact that the receipts had not fallen off more than was actually the case, the income from subscriptions having been £2,958 14s. 6d. instead of £3,053 15s. 4d. as in the previous year. At the same time, any loss was a serious thing, especially in view of the fact that for many years the society had been spending more than it received. He wished to emphasise one point, viz. that while the work of the society, originally confined to the City, now extended over the whole of the United Kingdom, and, indeed, all over the world, the support it received from the provinces was very small. He would like especially to appeal to provincial employers of labour to support a charity which rendered such timely aid to their workpeople, by restoring them to comparative comfort, and enabling them to pursue their avocations with safety. Alluding to the loss by death of several generous supporters, among whom was Mr. Alfred Lyon, a trustee for many years, the chairman said he hoped their places would be filled by new friends, thus enabling the committee to report next year a nearer approximation between the income and expenditure of the society.

Mr. Charles E. Keyser, a trustee, in seconding, prognosticated "better times," which would prevent this old charity from drawing upon invested capital to meet current expenses. If it were better known that only one letter was required for each case, he thought the number of supporters would inevitably increase. He noticed that the expenditure on advertisements had been reduced by £250; he hoped, in another year or two, that the society would be able to return to something like the amount formerly spent in that direction. The report and accounts were adopted.

The Rev. N. J. Devereux having suggested that an effort should be made to obtain provincial church collections, the meeting closed with the usual votes of thanks. Mr. John Langton, in returning thanks on behalf of the surgeons, referred to the kindness of the committee in allowing the staff a wide discretion in the matter of giving relief. He hoped the centenary in 1907 would be marked by a large increase in the number of subscribers to this invaluable charity.

